# Different pixel sizes of topographic data for prediction of soil salinity

**DOI:** 10.1371/journal.pone.0315807

**Published:** 2024-12-31

**Authors:** Shima Esmailpour, Ebrahim Mahmoudabadi, Mohammad Ghasemzadeh Ganjehie, Alireza Karimi

**Affiliations:** 1 Department of Soil Science, College of Agriculture, Ferdowsi University of Mashhad, Mashhad, Iran; 2 Department of Desert and Arid lands Management, Faculty of Natural Resources and Environment, Ferdowsi University of Mashhad, Mashhad, Iran; 3 Soil and Water Department, Khorassan Rzavi Agricultural and Natural Resources Research and Eduacation Center, Agricultural Research Education and Extention Organization (AREEO), Mashhad, Iran; Universidade Federal de Uberlandia, BRAZIL

## Abstract

Modeling techniques can be powerful predictors of soil salinity across various scales, ranging from local landscapes to global territories. This study was aimed to examine the accuracy of soil salinity prediction model integrating ANNs (artificial neural networks) and topographic factors with different cell sizes. For this purpose, soil salinity was determined at 103 points in the east of Mashhad, Razavi Khorasan, Iran. The region was categorized into two distinct parts: study area (1) (with a steep topography) and study area (2) (with a flat topography). To explore the impact of terrain on salinity prediction accuracy, ANNs were trained using topographical factors as inputs across a range of cell sizes (30, 50, 90, 200, and 500 m). The model’s effectiveness was evaluated based on their Root Mean Square Error (RMSE) and coefficient of determination (R^2^). Results indicated variability in model performance, with RMSE ranging from 0.324 to 0.461 and R^2^ from 0.159 to 0.281 across the spectrum of cell sizes. Deeper analysis on different topographical influences showed that for the study area (1), a cell size of 30 m yielded the most accurate predictions (RMSE = 0.234 dS/m and R^2^ = 0.515), whereas for the study area (2), a cell size of 50 m was optimal (RMSE = 0.658 dS/m and R^2^ = 0.597). In general, the findings concluded that smaller cell sizes can enhance prediction accuracy in areas with complex and varied topography, while larger cell sizes can be more effective in flat areas. This study demonstrates the significance of incorporating terrain attributes and their optimal resolutions for accurate soil salinity prediction. Our findings underscore the importance of tailoring the resolution of input data to match the specific topographic features of the area, challenging the conventional notion that higher input resolution invariably yields better results in soil properties prediction. These insights provide valuable guidance for effective soil management and agricultural practices, as well as contribute to more informed decision-making in land management and environmental conservation.

## Introduction

Soil resources provide a wide range of ecosystem services for regulating and supporting life on the earth. In some areas, human activities have led to soil salinization and caused many environmental issues particularly in arid and semi-arid regions. Moreover, soil salinization has been mentioned as one of the most important threats to sustainable agriculture production [1–5]. The first step in preventing and managing soil salinity is to have a precise and accurate map of the lands. Therefore, developing accurate models for mapping soil salinity is crucial for promoting sustainable crop farming and environmental health especially in regions with arid and semi-arid climates.

In recent years, digital soil salinity maps, owing to the provision of continuous maps, have been introduced as an alternative to the conventional salinity ones. This approach uses environmental data such as topographic factors to predict soil salinity [6–9]. For instance, Yahiaoui et al. [6] used topographic parameters for estimating the spatial distribution of soil salinity in the northwest Algeria. Mirzaee et al. [10] and Mirzaee and Ghorbani [11] employed topographic factors to improve the prediction accuracy of soil hydraulic properties in northwest of Iran. Peng et al. [7] and Wang et al. [8] applied the terrain factors to predict soil salinity in southern Xinjiang Province, China. Li et al. [9] mapped soil salinity by using topographic factors in the Yellow River Delta of China. Topographic parameters play important roles in controlling the distribution and redistribution of salts across different soil layers [12]. Steep landscapes generally can accelerate the movement of salt through the soil profile, while low-lying and flat landscapes may promote the accumulation of salt [7, 13].

Some researchers have reported the influences of the spatial resolution of topographic maps (i.e. cell size) on the accuracy of the produced maps. Thompson et al. [14] and Pain, [15] noted some potential limitations of using digital elevation models (DEM) with smaller cell sizes (higher resolutions) as input variables to identify earth objects. They suggested that the suitable spatial resolution of a DEM for areas with complex morphology involved using a smaller cell size, whereas a larger cell size could suffice for low-lying and flat areas.

The accuracy of a DEM depends on its spatial resolution which has a significant effect on the details captured in map information [16]. Although higher cell sizes (indicating lower resolutions) lead to the loss of critical details, particularly in areas characterized by valleys and hills, making the detection of variations in micro-topography a challenge [17]. Conversely, maps with smaller cell sizes (denoting higher resolutions) often contain an excess of details, making analysis laborious and time-consuming, particularly over extensive areas [17]. Moreover, the smaller cell sizes result in numerous small closed watersheds, creating various types of errors for analyzing topographic factors [18]. Several researchers have questioned the common assumption that higher resolution data invariably produces better results. Li and Zhou [19] discovered that intermediate-resolution data proved optimal for generating susceptibility maps when comparing different terrain data resolutions (ranging from 2 m to 300 m) in their models. Similarly, Lee et al. [20] observed negligible differences in map accuracy derived from DEM resolutions ranging from 5 to 30 m. While smaller cell sizes provide more detailed information, they may also hold an excess of details and present technical and computational challenges [21]. As spatial resolution increases, the size of the raster expands by the square of the increase in resolution, making it critical to control resolution to achieve the intended map scale without compromising data processing efficiency. Although numerous studies have highlighted terrain attributes such as elevation [6], topographic wetness index (TWI) [22], DEM [8], channel network (VDCN), and analytical hill-shading (AH) [23] as the most effective covariates among various auxiliary information for salinity prediction, only a few researches have investigated how different pixel sizes in topographic maps as covariates affect the accuracy of predictive models of soil properties [14, 15]. On the other hand, topography significantly influences soil salinity through its control over hydrological processes, including the movement and accumulation of water and dissolved salts. For example, slope and elevation factors dictate the direction and speed of surface runoff and groundwater flow, affecting the distribution of salts [1, 6]. Therefore, the main objectives of the present study were to: (i) develop artificial neural networks (ANNs) to predict soil salinity using topographic factors with different spatial resolutions for whole study area, and (ii) evaluate the performance of ANNs models in two distinct regions located in the eastern part of Mashhad, Razavi Khorasan province, Iran. These regions include an area with steep topography (study area (1)) and another with a predominantly low-lying and flat terrain (study area (2)).

## Materials and methods

### Study region

**Fig 1:**
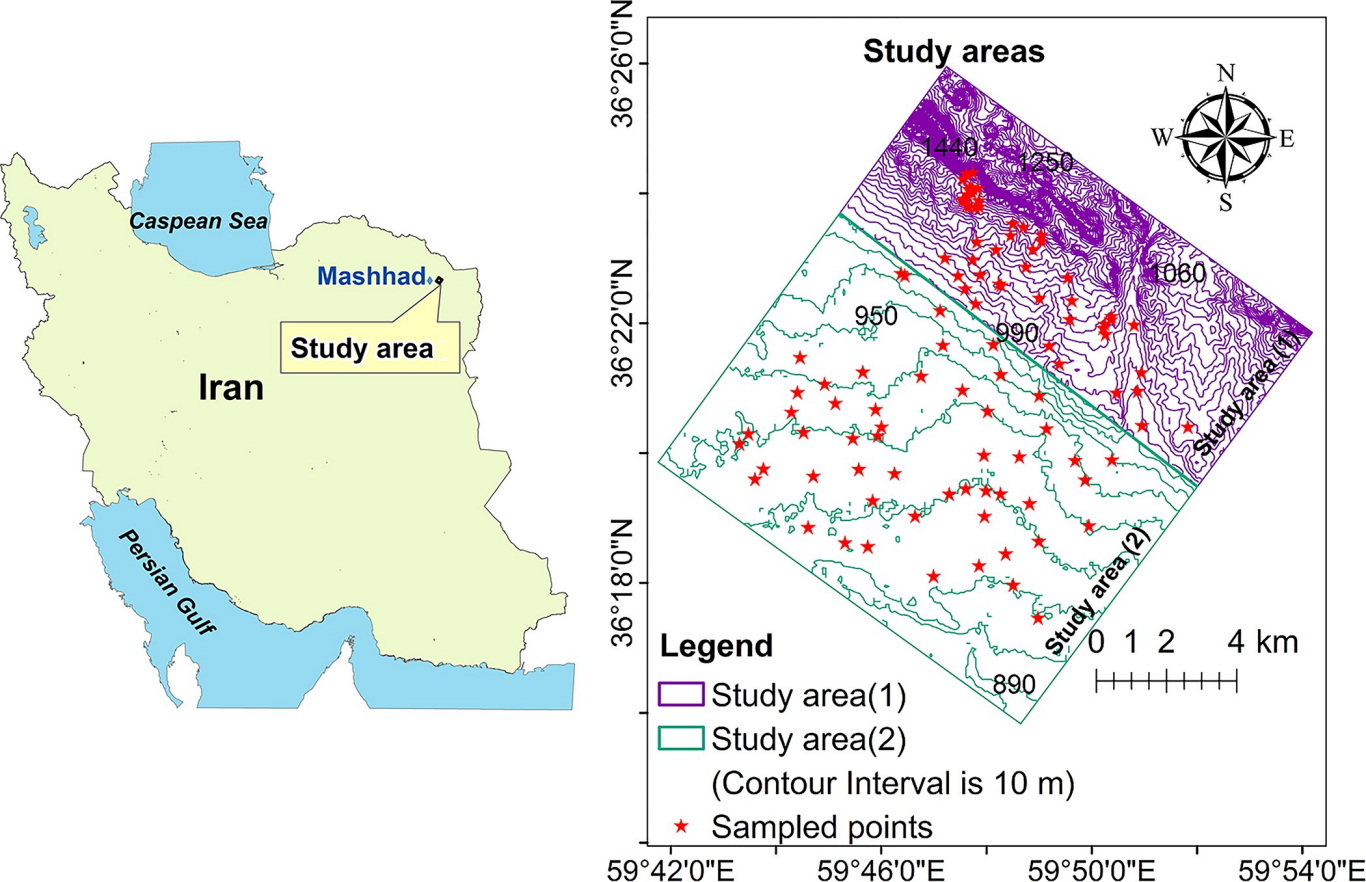
Distribution of the taken samples in the studied regions.

**Fig 2:**
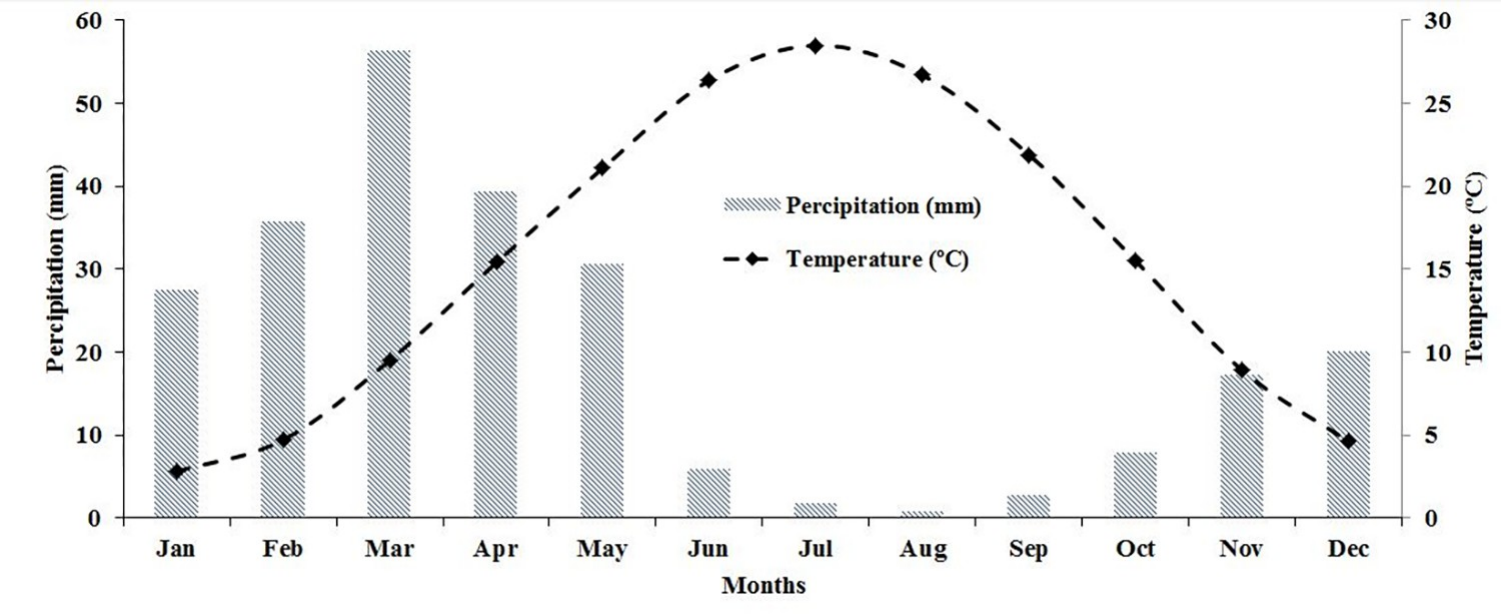
The monthly mean temperatures and precipitation (World Meteorological Organization, from 1991 to 2020).

**Fig 3:**
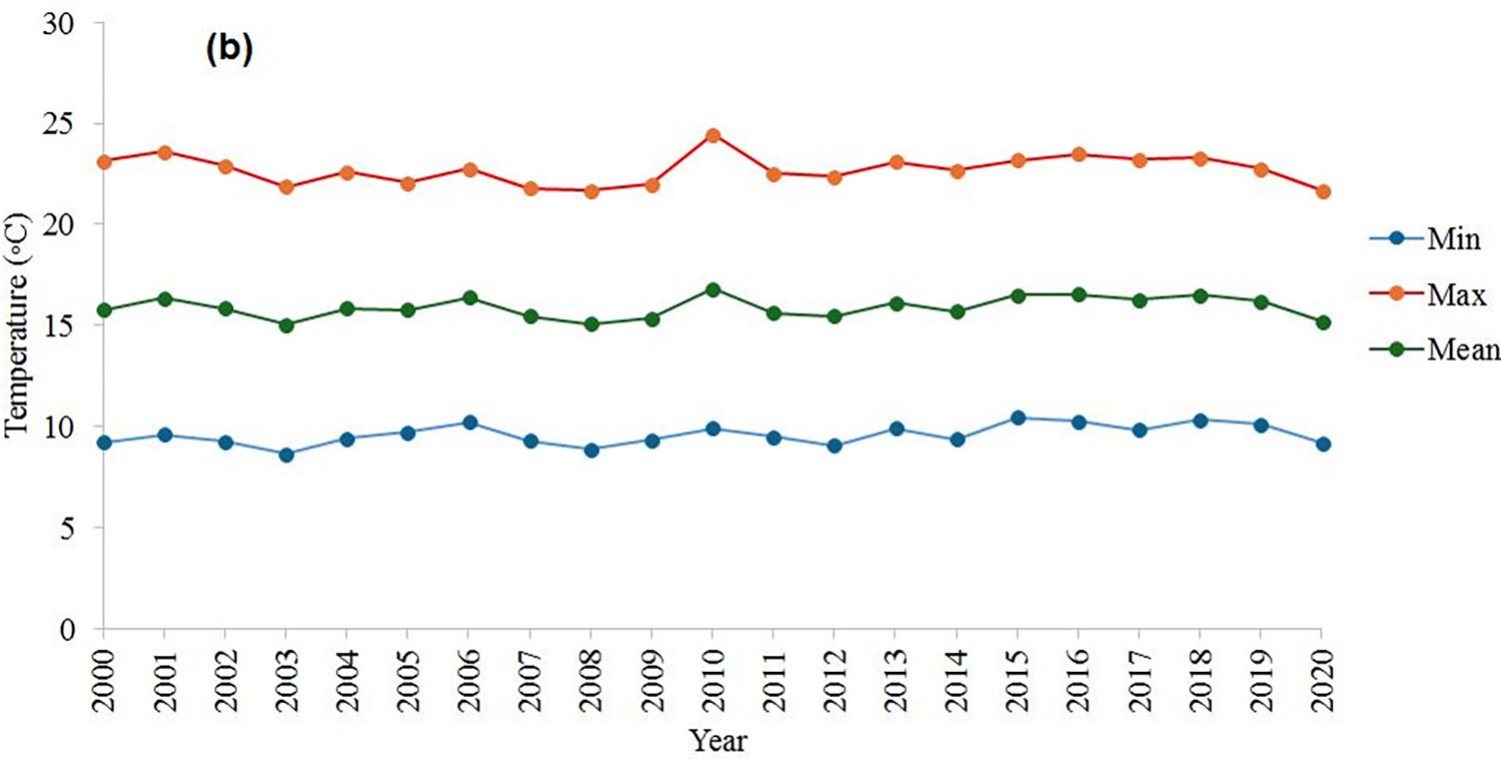
The annual minimum, maximum and mean temperature variations for the study area (World Meteorological Organization, from 2000 to 2020).

According to Fig 1, the study was conducted in the east of Mashhad, Razavi Khorasan province, Iran with longitude 36° 15.83′ to 36° 26.03′ N and latitude 59° 42.88′ to 59° 54.18′ E. This region covered approximately 17743 ha. The main agriculture crops in this area are generally maize, wheat, sugar beet and barley. Most native plant species have died out as a result of intensive human activities. Alluvial soils in the southern part of the study region are typically formed by the deposition of sediments carried by surface runoff. Based on the soil taxonomy classification, the study area contained two soil orders: Entisols and Aridisols [24], with the former was primarily found in the northern and eastern parts and the latter was predominantly located in the southern and western parts of the study region. Precipitation and temperature data recorded by World Meteorological Organization for 1991 to 2020 are presented in Figs 2 and 3. As can be seen from Fig 2, the mean monthly temperatures ranged from 2.8 to 28.5°C. Monthly precipitation varied from 0.8 to 56.3 mm, with an annual mean of 245.8 mm (Fig 2).

### Topographic factors

A digital elevation model (DEM) is a representation of the topography of a surface. In this study, a DEM with a resolution of 30 m was obtained from the National Cartographic Center of Iran (NCC). Before calculating terrain parameters, it is crucial to detect and minimize errors in the DEM to ensure the accuracy of the terrain parameters calculated [25].

#### Errors detection and quantification

The initial errors which are required to be reduced are padi terraces or cut-offs. The padi terraces or cut-offs areas are characterized by adjacent pixels displaying identical values and can be described as:

$$e\leftarrow [\forall c\;{zNB}_{c}=z{NB}_{x}]$$ (1)

Where z is the value of DEM, e is the error, $z{NB}_{x}$ is the value of the central cell and ${zNB}_{c}$ is the value at the *c*th neighbor of its *k × k* window. Padi terraces phenomenon increases due to the omission of hilltops and small ridges, and valley bottoms are typically not recorded in topo-maps. Detecting padi terraces in a GIS can be achieved through the application of a neighborhood operation.

The second type of error that should be identified is local outliers, which are characterized as small, highly improbable anomalies that can be increased by significant errors in the data collection process, particularly in cases involved using remote sensing instruments where such errors are more prevalent. Local outliers can be detected and quantified using the statistical method proposed by Felicísimo, [26]. This approach involves calculating the probability of finding a specific value within the neighborhood by comparing the original values with the estimated values derived from neighboring data points.


$$\delta_{i}={\hat{z}}_{i}^{NB}-z_{i}$$ (2)

Where *δ*_*i*_ is the difference between the predicted and original values. ${\hat{z}}_{i}^{NB}$ shows the values predicted from the neighbors.

#### Errors reduction

Improving the quality of the DEM is crucial primarily to reduce the outliers. By minimizing the presence of the outliers, a smoothed DEM can be obtained through a weighted average of the original DEM and estimated values. This process contributes to an improved and more reliable representation of the DEM as follows:

$$Z_{i}^{+}=P(t_{i})\times z_{i}+[1-P(t_{i})]\times {\hat{z}}_{i}^{NB};\;P(t)\in [0.1]$$ (3)

Where z^+^ represents the DEM value. The p(t) shows the probability of surpassing a value predicted from neighboring data points by employing the spatial dependence structure. The next step to improve DEM is the adjustment of sink in the original DEM. The sink in original DEM was adjusted by the following equation [17]:

$$\Delta Z_{i}={\left(\frac{P}{P_{i}+d_{i}}\right)}^{\varphi }H;\;\Delta Z_{i}\in [0.H]$$ (4)

$$Z_{i}^{+}=z_{i}+\Delta Z_{i}$$ (5)

Where *H* is the max of DEM value difference, *P* represents pixel size, *ϕ* indicates adjustment factor, *d*_*i*_ shows the distance to a stream and Δ*Z*_*i*_ shows the adjustment of DEM value.

#### Topographic factors in the present study

**Fig 4:**
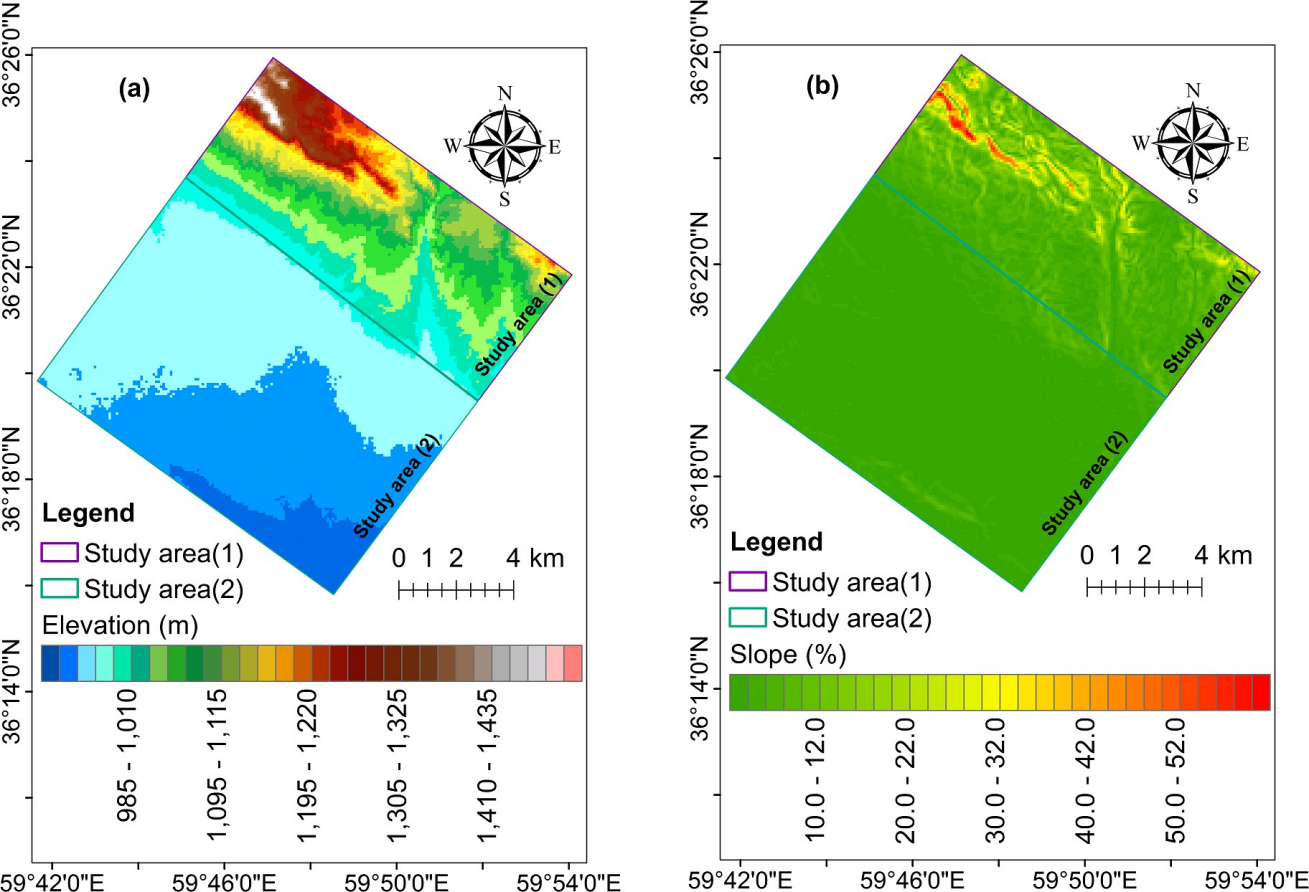
Topographic factors of the study areas: elevation (a) and slope (b).

Topographic factors were generated from Digital Elevation Models (DEMs) using ArcGIS v. 10.3 (Table 1). Drawing on the findings from a literature review [6–11], we chose DEMs with a range of cell sizes (30, 50, 90, 200, and 500 m) to capture terrain attributes. Various ArcGIS toolboxes were employed to extract or calculate factors from the DEM map, including elevation, slope, aspect, profile curvature, plan curvature, and catchment area. The "Raster Calculator" tool was used to compute additional factors such as flow accumulation, topographic wetness index, length-slope factor, stream power index, and sediment transport index. The primary topographic factors such as elevations in the entire study area, study area (1) and study area (2) varied from 882 to 1516 m, 957 to 1516 m and 882 to 992 m, respectively (Fig 4). Moreover, the slope factor varied from 0.0 to 62.2, 0.0 to 62.2 and 0.0 to 25.3% in the entire study area, study area (1) and study area (2), respectively (Fig 4).

**Table 1:** Definitions, formulas and significances of the selected topographic factors.

Variables	Definition	Formula	References
Elevation (h)	Height from see level	―	
Slope (S) (radian)	An angle between a tangent and a horizontal plane at a given point	$S=arctan\sqrt{p^{2}+q^{2}}$	[27]
Aspect (A) (radian)	An angle clockwise from north to the horizontal projection of an external normal vector at a given point	$A=arctan(\frac{q}{p})$	[28]
Profile curvature (m ^-1^)	Curvature of the surface in the direction of the steepest slope	$k_{v}=-\frac{p^{2}r+2pqs+p^{2}t}{(p^{2}+q^{2})\sqrt{1+p^{2}+q^{2}}}$	[29]
Plan curvature	Curvature in a horizontal plane	$k_{h}=-\frac{q^{2}r+2pqs+q^{2}t}{(p^{2}+q^{2})\sqrt{1+p^{2}+q^{2}}}$	[29]
Catchment area (m^2^)	Area draining to the catchment outlet	―	[27]
Flow accumulation (FA)	Upslope number of grid cells	―	[10]
Topographic wetness index (TWI)	Frequencies and duration of saturated conditions	$TWI=\ln\left(\frac{A_{S}}{S}\right)$	[30]
Length-Slope factor (LS)	Erosive power of the terrain	$LS=(n+1)STI={\left(\frac{A_{S}}{22.13}\right)}^{0.4}{\left(\frac{\sin\theta }{0.0}\right)}^{1.3}$	[27]
Stream power index (SPI)	Erosive power of overland flow	*SPI* = *A*_*s*_ tan *θ*	[30]
Sediment transport index (STI)	Soil-water content and sediment transport	$STI={\left(\frac{A_{S}}{22.13}\right)}^{0.4}{\left(\frac{\sin S}{0.0896}\right)}^{1.3}$	[30]

Where p, q, r, and t are partial derivatives of elevation (h): $h=f(x.y);p=\frac{\delta h}{\delta x};q=\frac{\delta h}{\delta y};r=\frac{\delta^{2}h}{{\delta x}^{2}};t=\frac{\delta^{2}h}{{\delta y}^{2}}$

### Soil sampling and analysis

Collecting soil samples is an important step in soil analysis. In this study, a total of 103 soil samples were collected from the root zone of most plants (about 0–30 cm) in June 2022. The studied regions were sampled by employing the elevations and slope classes in a stratified random sampling method. After that, the samples were air-dried to remove excess moisture and prevent microbial activity. Then, the samples were sieved using a 2 mm-sieve. The soil organic carbon content of the soil samples was analyzed using the wet-oxidation method developed by Nelson and Sommers, [31]. The hydrometer method, developed by Gee and Bauder [32], was employed to measure soil particles (clay, silt and sand contents). The back-titration method, developed by Nelson and Sommers, [31], was used to determine calcium carbonate equivalent (CCE). Electrical conductivity (EC) was determined in the extracted solution from the saturated soil [33].

In order to rescale both the target data and the input data, Eq 6 was applied as follows:

$$X_{n}=0.1+0.8\left[\frac{X-X_{min}}{X_{max}-X_{min}}\right]\;X_{min}<X<X_{max}$$ (6)

Where X_n_ represents the data points that have the rescaled data, while X_min_ and X_max_ correspond to the minimum and maximum values in the original dataset.

### Modeling structures

A feed-forward artificial neural networks (ANNs) with a multilayer perceptron architecture, a type of machine learning model, has been used in many soil science studies [34–36]. The MLP architecture consists of an input layer, one or more hidden layers, and an output layer. In addition, each layer consists of a set of neurons that are inter-connected by weights and are adjusted during the training process to optimize the performance of the developed model. The backpropagation learning rule is a widely used algorithm for training feed-forward ANNs with multilayer perceptron MLP architecture. It is an iterative algorithm that adjusts the weights and biases of the network to minimize the error between the estimated and observed data [37]. The backpropagation learning rule is used to train the MLP neural network. This learning rule involves iteratively adjusting the weights and biases of the network in order to minimize the prediction error using a set of training examples. This allows the network to learn the relationships between the input data and the target output variable [37].

### Input variables for developing ANNs model

**Fig 5:**
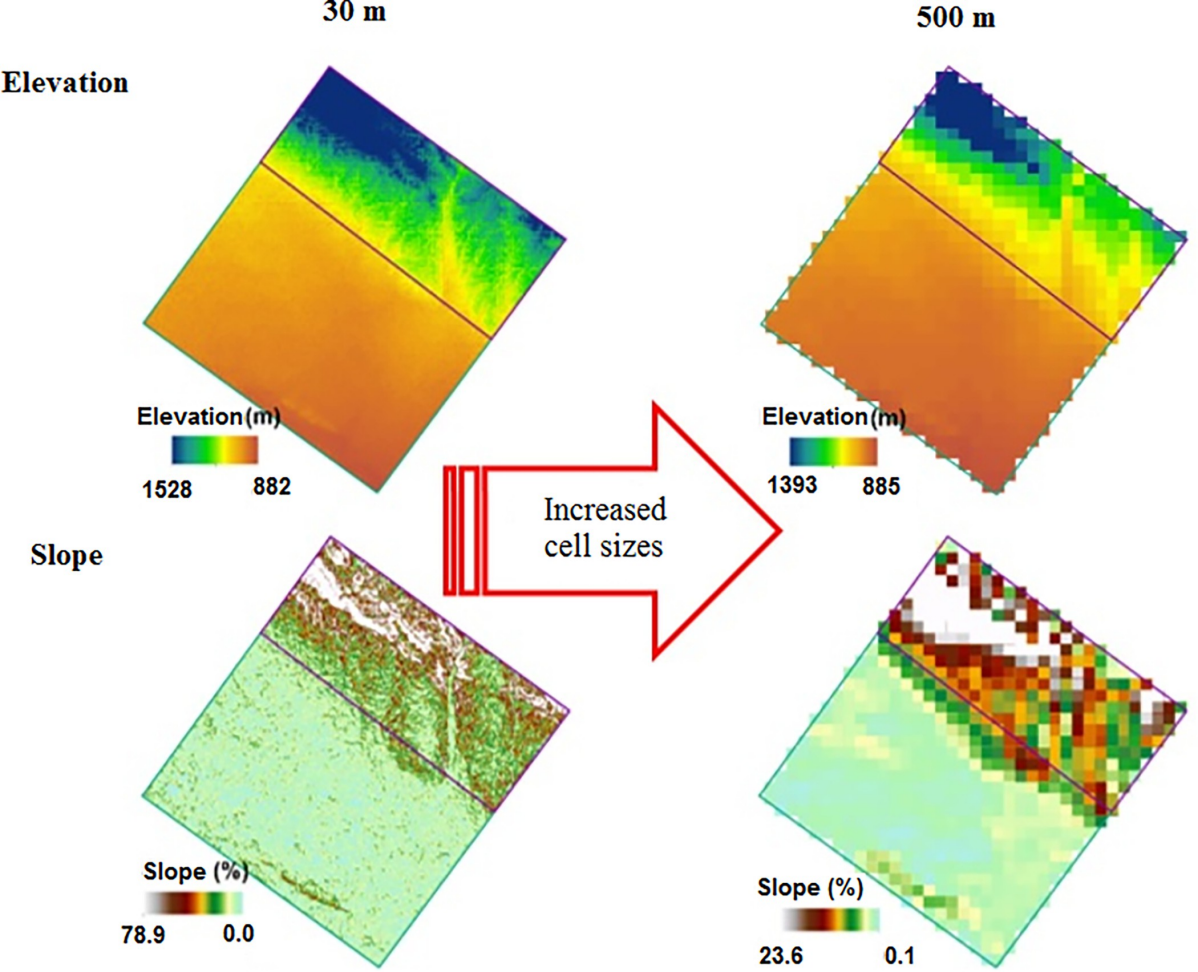
The maps of elevation and slope factors at different cell sizes.

**Fig 6:**
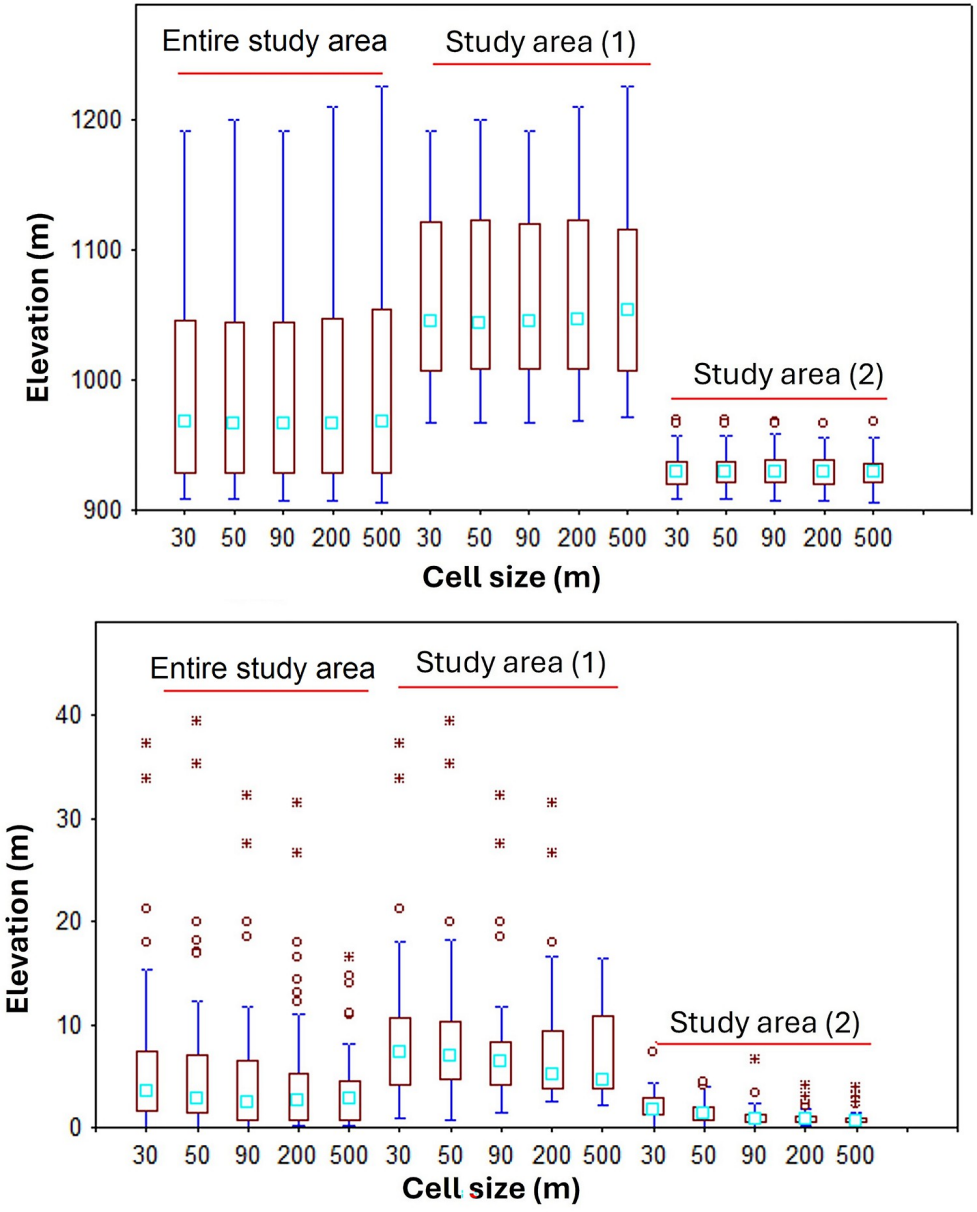
The data of elevation and slope factors at different cell sizes in the entire study area, study area (1) and study area (2).

Topographic factors (Table 1) including those with different cell sizes of 30, 50, 90, 200 and 500 m acquired from a DEM were introduced as input data in the models. As an example, maps displaying slope and elevation factors at the resolutions of 30 and 500 m are presented in Figs 5 and 6 illustrate the input variables with different spatial resolutions and different datasets including the data point located in the entire study area, study area (1) and study area (2). As can be seen from Fig 6, the values of slope and elevation factors are higher in the study area (1) than the study area (2), indicating that the sample points in the study area (1) feature steep terrain, while the study area (2) is characterized by low-lying and flat terrain.

### Model evaluation

In machine learning methods such as ANNs, error processing and model evaluation are important. For this reason, data in the entire study area, study area (1) and study area (2) were divided randomly into train and test data sets, with the former containing 70% of the data and the latter containing 30% of the data. To statistically evaluate the developed models, the following statistical indices were applied:

$$ME=\frac{1}{N}\sum_{i=1}^{N}(\hat{{EC}_{i}}-{EC}_{i})$$ (7)

$$RMSE={\left[\frac{\sum_{i=1}^{N}{\left(\hat{{EC}_{i}}-{EC}_{i}\right)}^{2}}{N}\right]}^{0.5}$$ (8)

$$R^{2}=1-\frac{\sum_{i=1}^{N}{\left({EC}_{i}-\hat{EC}\right)}^{2}}{\sum_{i=1}^{N}{\left({EC}_{i}-\bar{{EC}_{i}}\right)}^{2}}$$ (9)

$${RI}_{x}=\frac{{RMSE}_{x}-{RMSE}_{ref}}{{RMSE}_{ref}}\times 100$$ (10)

Where ME is the mean error, RMSE is root mean squared error and R^2^ is the coefficient of determination. $\hat{{EC}_{i}}$ is the estimated electrical conductivity data, EC_*i*_ is the measured electrical conductivity data, $\bar{{EC}_{i}}$ is the average of measured electrical conductivity data and N is the number of sample points. RMSEx represents the RMSE value calculated for the x method, while RMSE_ref_ corresponds to the RMSE value obtained for the reference method. The reference method in this study was the RMSE obtained using topographic factors with a 500 m cell size as input variables.

## Results and discussion

### Soil attributes

**Fig 7:**
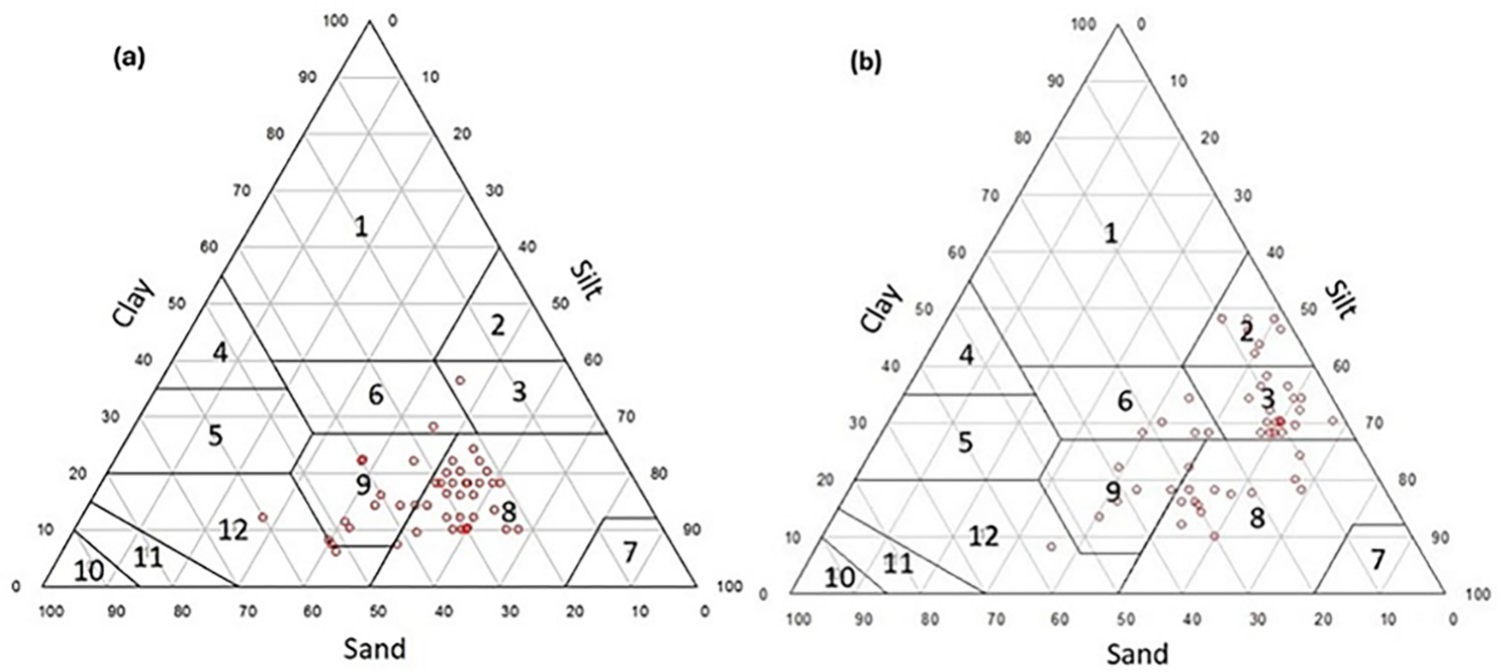
USDA soil textural distribution for study area (1) (a) and study area (2) (b). 1: Clay, 2: Silty clay, 3: Silty clay loam, 4: Sandy Clay, 5: Sandy clay loam, 6: Clay loam, 7: Silt, 8: Silt loam, 9: Loam, 10: Sand, 11: Loamy sand, and 12: Sandy loam).

The descriptive statistics for sample points located in the entire study area, study area (1) and study area (2) are shown in Table 2. Soil particles showed a wide range of distribution in all the studied areas. For example, clay contents in the entire study area, study area (1) and study area (2) ranged from 6.0 to 48 (CV = 48.62%), 6.0 to 36.1 (CV = 37.80%) and 8.0 to 48.0% (CV = 38.84%), respectively. The soil texture classes are shown in Fig 7. Soil texture classes in the study area (1) were silty clay loam (1.97%), clay loam (1.97%), loam (25.50%), sandy loam (3.94%), and silt loam (66.85%). Textural classes of the soils in the study area (2) were silty clay (13.46%), silty clay loam (36.69%), clay loam (9.61%), silt loam (27.00%), loam (11.53%), and sandy loam (1.92%). Soil organic carbon varied from 0.42 to 1.41 (CV = 26.07%), 0.42 to 1.41 (CV = 29.65%) and 0.51 to 1.32% (CV = 22.45%) for samples of the entire study area, study area (1) and study area (2), respectively (Table 2). Moreover, calcium carbonate equivalent indicated high variability in the region (i.e. a CV = 34.04, 43.73, and 22.43% for abovementioned areas, respectively (Table 2)).

**Table 2:** Soil properties data for the entire sample points, study area (1) and study area (2).

Study area part	Soil properties	Min	Max	Mean	St.D	CV (%)
The entire study area	Clay (%)	6.0	48.0	21.35	10.38	48.62
	Silt (%)	28.0	69.3	53.07	8.47	15.95
	Sand (%)	1.8	60.0	25.58	13.47	52.65
	pH	6.75	8.04	7.37	0.22	2.98
	Organic carbon (%)	0.42	1.41	0.78	0.20	26.07
	Calcium carbonate equivalent (%)	5.62	46.01	23.95	8.15	34.04
Study area (1)	Clay (%)	6.0	36.1	15.38	5.81	37.80
	Silt (%)	28.0	68.0	52.2	8.94	17.13
	Sand (%)	17.8	60.0	32.3	10.25	31.65
	pH	6.88	8.04	7.39	0.21	2.81
	Organic carbon (%)	0.42	1.41	0.76	0.23	29.65
	Calcium carbonate equivalent (%)	5.62	46.00	23.28	10.18	43.73
Study area (2)	Clay (%)	8.0	48.0	27.2	10.57	38.84
	Silt (%)	36.0	69.3	53.89	7.96	14.77
	Sand (%)	1.8	56.0	18.9	12.94	68.52
	pH	6.75	7.89	7.36	0.23	3.15
	Organic carbon (%)	0.51	1.32	0.79	0.18	22.45
	Calcium carbonate equivalent (%)	10.87	37.50	24.61	5.52	22.43

### Measured target variable (soil salinity)

**Fig 8:**
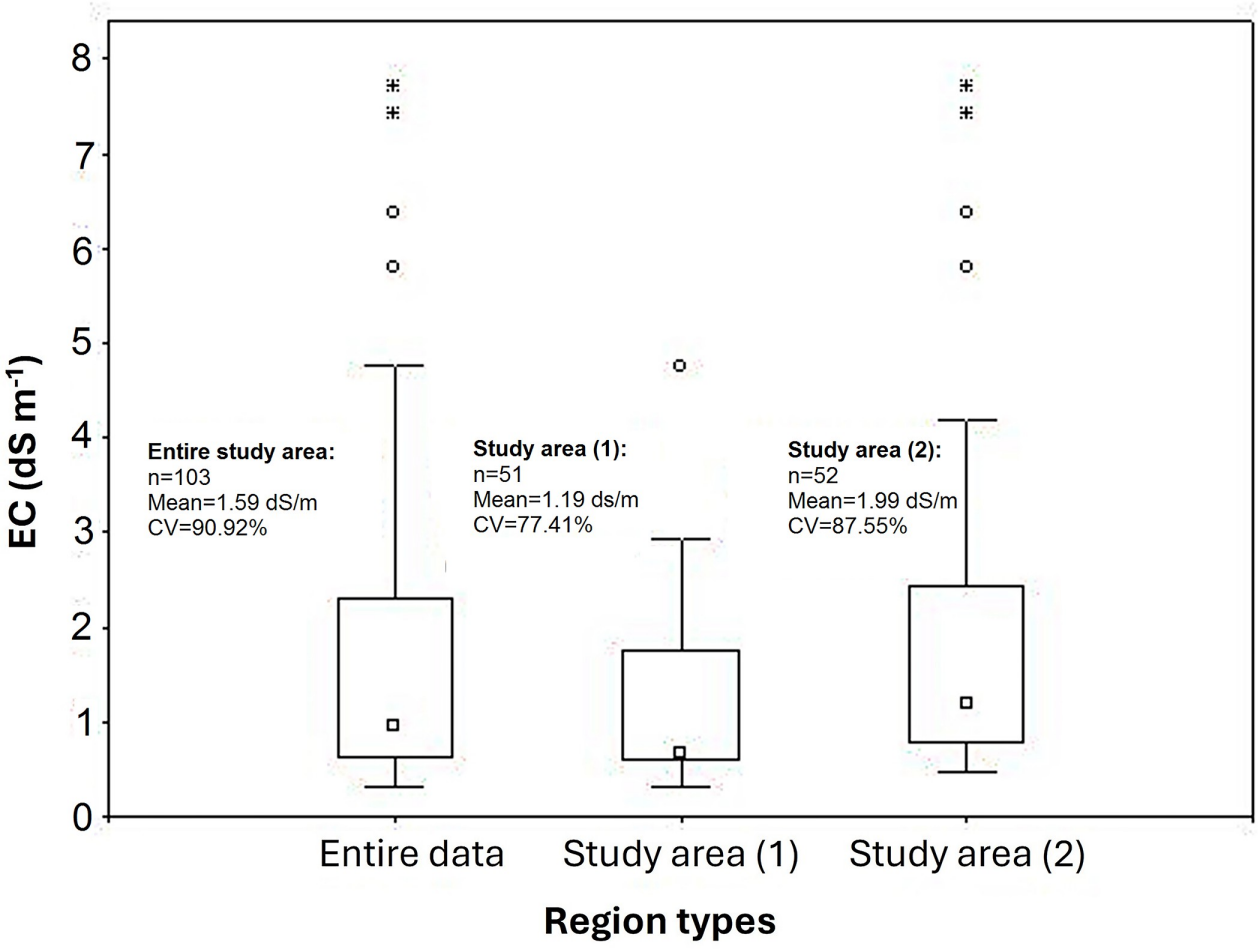
EC data for the entire samples points, study area (1) and study area (2).

Fig 8 shows EC values for the entire measured data, as well as the study areas (1) and (2). The average measured EC data for the entire points were 1.59 dS m^-1^ with a range of 0.32 to 7.71 dS m^-1^ (Fig 8). For the study areas (1) and (2), the mean measured EC data were 1.19 dS m^-1^ with a range of 0.32 to 4.76 dS m^-1^, and 1.99 dS m^-1^ with a range of 0.46 to 7.71 dS m^-1^, respectively (Fig 8). The measured EC values of the study area (1) were lower than those of the study area (2) (Fig 8), which can be owing to steep slope situation and high variation in topography of the study area (1). In addition, soils in this area were mostly shallow and classified as Entisols with lighter texture. For this reason, soil salts were carried away by surface flow and leaching processes to the lowland regions such as study area (2). Similarly Fan et al. [13] reported that various factors are involved in soil salinization in the Yellow River Delta (YRD). It is also reported that geographic and geomorphic conditions make prominent contribution to distribution and redistribution of salt. Compared to regions with high steep slope which lead to salinity movement, the regions with low-lying landscape in the YRD increased the accumulation of salt. Furthermore, the high CV values (i.e. 90.92% for the data of the entire study area, 77.41% for study area (1) and 87.55% for study area (2)) are important features to develop some reliable models for predicting soil salinity.

### Evaluation of the developed ANNs using the data of the entire study area

In this research, ANN models were derived by employing topographic factors with different spatial resolutions as input variables. The results of the developed ANN-based models with different cell sizes are shown in Table 3 for the entire study area. As for the entire study area, employing topographic factors with the test data set of 50 m cell size resulted in the smallest RMSE (0.324 dS m^-1^) and the highest R^2^ value (0.281). In addition, the model developed using topographic factors with 50 m cell size, based on the ME criteria, indicated a tendency to over-estimate soil salinity (ME = 0.3120 dS m^-1^) (Table 3).

**Table 3:** Performance of ANN models using test data sets with different cell sizes for the entire study area.

Cell size	Selected architecture	Transfer functions	ME	RMSE	R^2^
30	2-4-1	logsig-purelin	-0.3149	0.344	0.267
50	2-9-1	logsig-purelin	0.3120	0.324	0.281
90	2-6-1	logsig-purelin	0.0019	0.350	0.224
200	2-6-1	tansig-Purelin	0.0097	0.346	0.244
500	2-6-1	logsig-purelin	-0.0273	0.461	0.159

### Evaluation of the developed ANNs for study area (1)

Results related to the derived models with different cell sizes for the study area (1) are presented in Table 4. Employing topographic factors with different spatial resolutions for estimating EC in high steep region (i.e. study area (1)) indicated that ME values ranged from -0.0473 to 0.0428 dS m^-1^ in testing phase (Table 4). Moreover, RMSE and R^2^ with different cell sizes for the study area (1) are shown in Table 4. The performance of the developed models showed that the ANN model developed using topographic factors with 30 m cell size was the best (RMSE = 0.234 dS m^-1^ and R^2^ = 0.515 in test data set) (Table 4).

**Table 4:** Performance of ANN models using test data sets with different cell sizes for the study area (1).

Cell size	Selected architecture	Transfer functions	ME	RMSE	R^2^
30	2-9-1	logsig-purelin	0.0000	0.234	0.515
50	2-9-1	tansig-Purelin	-0.0473	0.269	0.323
90	2-6-1	logsig-purelin	0.0428	0.382	0.267
200	2-5-1	logsig-purelin	0.0000	0.388	0.230
500	2-5-1	logsig-purelin	0.0000	0.410	0.212

### Evaluation of the developed ANNs for study area (2)

The performance of the models developed using topographic factors as input variables for the study area (2) is presented in Table 5. Comparison of the values of statistical criteria including ME, RMSE and R^2^ in Table 5 indicated that the best performance (ME = 0.0879 dS m^-1^, RMSE = 0.658 dS m^-1^, and R^2^ = 0.597) for predicting soil salinity in the study area (2) obtained by using 50 m cell size, followed closely by 90 m cell size (ME = -0.0433 dS m^-1^, RMSE = 0.684 dS m^-1^, and R^2^ = 0.594) (Table 5). However, employing topographic data with 500 m cell size resulted in the highest RMSE and the lowest R^2^ values (Table 5).

**Table 5:** Performance of ANN models using test data- sets with different cell sizes for the study area (2).

Cell size	Selected architecture	Transfer functions	ME	RMSE	R^2^
30	2-6-1	logsig-purelin	-0.0485	0.701	0.561
50	2-8-1	logsig-purelin	0.0879	0.658	0.597
90	2-8-1	logsig-purelin	-0.0433	0.684	0.594
200	2-4-1	tansig-Purelin	-0.0274	0.913	0.430
500	2–81	tansig-Purelin	0.0089	0.980	0.346

### Evaluation of the developed ANNs models to predict soil salinity

**Fig 9:**
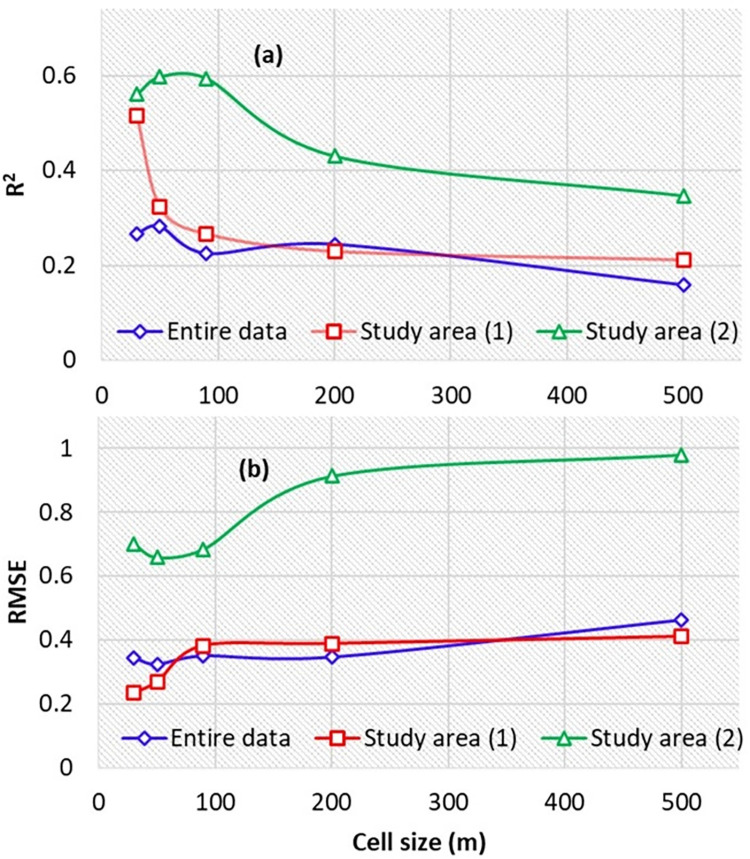
Estimation accuracy by using topographic factors as input variables in different cell sizes.

**Fig 10:**
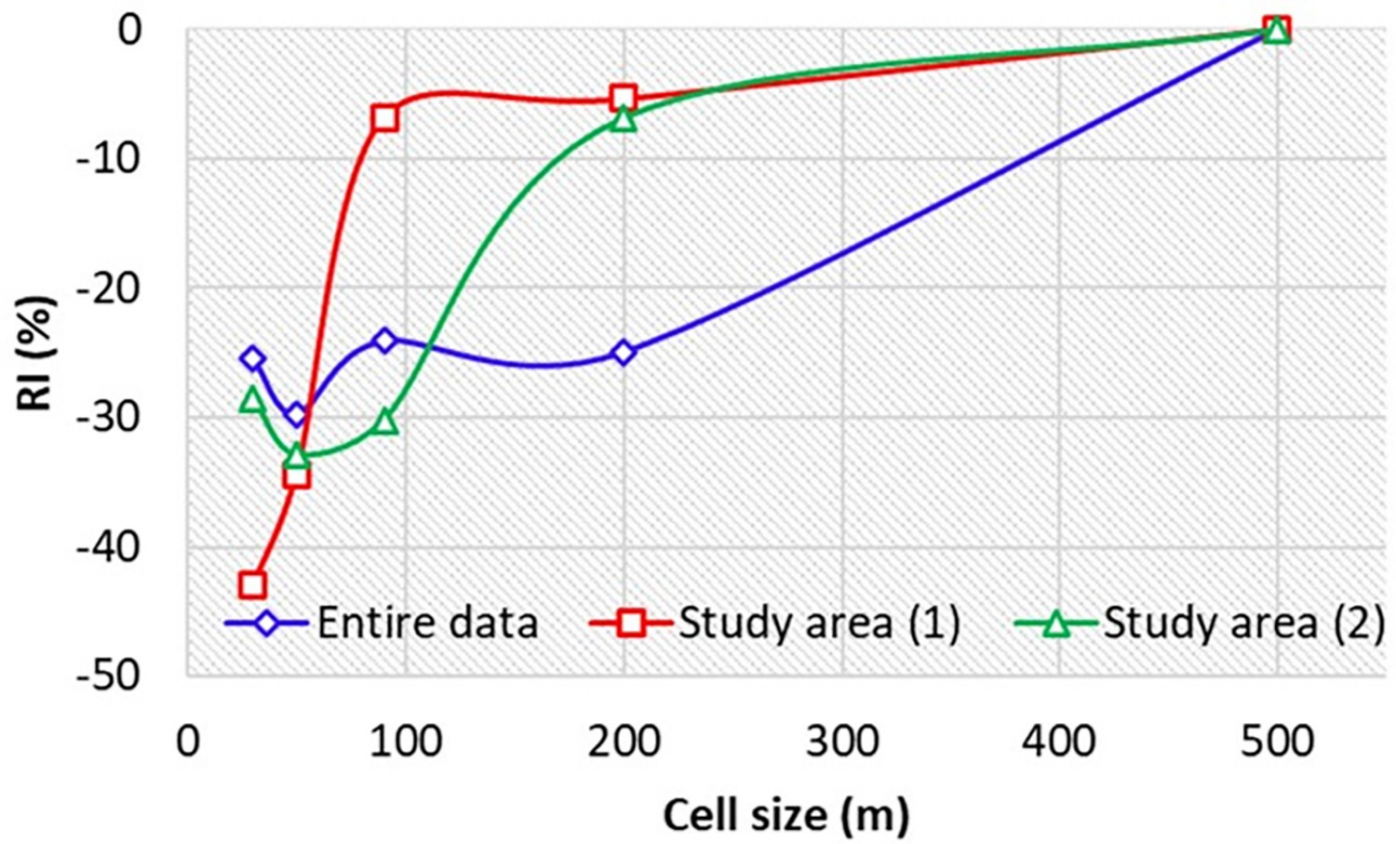
The relative accuracy improvement of the developed methods with different cell sizes relative to the reference method (the results of the model with 500 m cell size).

In the current research, in order to improve the performance of the derived ANN models, topographic factors derived from DEMs with different cell sizes were used in two different topographical conditions. The results presented in Fig 9 showed that soil salinity prediction performances by ANN models varied with topographic data in different spatial resolutions. In addition, as can be seen from Fig 9, the model performances were varied depending on topographical conditions (i.e. study area (1) with high steep topographic situations and study area (2) with low-lying and flat topographic situations). Therefore, the status of the region topography and consequently classifying the region into two parts including study area (1) with high steep topographic situations and study area (2) with low-lying and flat topographic situations influenced the performance of the developed ANN models. The results showed that the best cell sizes of topographic factors for predicting soil salinity for the entire study area, study area (1) and study area (2) were 50, 30 and 50 m, respectively (Fig 9). According to these results, there is no need to lower cell sizes in low-lying and flat topographic situations such as the study area (2). It could be related to recording unnecessary data, such as the creation of numerous small closed watersheds, which lead to errors in topography analysis [18]. By contrast, in high steep topographic situations, there is a need to use lower cell sizes (higher resolutions) to identify micro-topography variations, valleys and hills. In addition, application of topographic factors with different cell sizes, such as 30, 50, 90 and 200 m, resulted in a higher relative improvement (RI) compared to the use of topographic factors with 500 m resolution (Fig 10).

Several researches have reported that using topographic factors can improve the model accuracy [38, 39]. For instance, Ren et al., [39] reported that soil salinity distribution is controlled by topography and groundwater depth. Generally, soil salinity values are high in flat areas and reduce with increasing elevation factors [40, 41]. Elevation effects can be related to the higher precipitation, which consequently increases the possibility of more vegetation and transfers salts to the low-lying and flat topographic areas by surface flow and leaching. Elevation influenced soil moisture, which plays an important role in the distribution of soil properties [42]. Areas situated at lower elevations tend to be warmer than those at higher elevations, promoting higher rates of evaporation [43]. This increased evaporation facilitates the accumulation of soluble salts at soil surface. Similarly, flat areas are more susceptible to strong local winds, which further enhance evaporation and contribute to the accumulation of salts at soil surface [44]. Moreover, slope degrees have a great effect on the distribution of soil properties such as soil texture, organic matter, and salinity [45]. This relationship is pivotal in understanding how various factors such as solar radiation, aspect, and hydrology can affect soil dynamics and vegetation patterns. Specifically, solar radiation received in north-facing aspects is low, which affects soil properties and vegetation growth [46, 47] due to changes in soil water content and temperature. Differences in hydrological and received solar energy linked to aspect may induce the divergence in vegetation intensity, soil formation, and the risk of soil salinization, leading to local variations in temperature and precipitation, and acting as critical determinants of soil property diversity [48–50]. In addition, in inundated areas, Topographic wetness index (TWI) is an effective attribute for investigating soil-water content spatial variation along the slope [51]. TWI is used to determine soil moisture gradients, serving as a valuable indicator showing the potential of alterations in soil properties. The findings of Liu et al. [51] highlighted the significance of TWI as a key terrain attribute influencing soil characteristics. Several studies, such as those reported by Mirzaee et al. [10]; Ostovari et al. [52]; Tashayo et al. [53]; Wang et al. [8] and Li et al., [9], have employed topographic factors alongside a range of cell sizes from 30 to 500 m to predict various soil properties. Such researches underscore the profound impacts of topographical features on soil conditions, emphasizing the need for incorporating topographic considerations into soil properties assessments and environmental management strategies. This comprehensive approach enables a more in-depth understanding of soil behavior, facilitating effective land use planning and agricultural practices tailored to the specific needs of the terrain. Despite the efficacy of employing topographic factors with varying cell sizes for soil salinity prediction, it is crucial to recognize certain limitations of this study. Our findings suggest that optimal terrain attribute resolutions were 50 m for flat terrain and 30 m for steep terrain, highlighting the importance of selecting covariate resolutions that align with the scale of variation in the predicted soil property. As a result, different fields with different salinity variations may require unique optimal covariate resolutions. These considerations may affect the generalizability of the study’s findings, as the optimal cell sizes identified for this specific region might not be directly applicable to other areas with distinct topographical features and salinity variations. Furthermore, while this study concentrated on employing Artificial Neural Networks (ANNs) for soil salinity prediction, it would be beneficial to examine the performance of ANNs in comparison to other machine learning methods, such as Random Forest, Support Vector Machines, and Gradient Boosting Machines. By incorporating such comparisons in future research, a more comprehensive understanding of the predictive capabilities and limitations of ANNs relative to other techniques can be gained. This broader analysis would provide valuable insights into the strengths and weaknesses of ANNs in the context of soil salinity prediction and contribute to the development of more accurate and efficient models.

## Conclusion

Soil salinity was predicted using an ANN model and topographic factors with different cell sizes (from 30 to 500 m) in the east of Mashhad, Razavi Khorasan province, Iran. The study region was divided into two parts including study area (1) (with steep topographic situations) and study area (2) (with low-lying and flat topographic situations). The results of the present research showed that the best cell sizes of topographic factors for predicting soil salinity for the entire study area, study area (1) and study area (2) were 50, 30 and 50 m, respectively. According to the obtained results, the higher cell sizes (lower resolutions) are recommended for low-lying and flat topographic situations such as the study area (2), while the lower cell sizes (higher resolutions) are recommended for steep topographic situations such as the study area (1). However, topographic factors were the most important environmental variables affecting the soil properties. Future studies can further explore the relationship between cell size and prediction accuracy by testing the model across diverse landscapes and climatic conditions. This would provide a more comprehensive understanding of how cell size impacts prediction performance in various contexts. Additionally, assessing the performance of different machine learning models with varying input resolutions in regions with more diverse land use or vegetation cover could help establish the broader applicability of these findings. Such research would contribute to the development of more accurate and efficient models for soil salinity prediction across a range of environmental conditions.
